# Extraction vs. Nonextraction on Soft-Tissue Profile Change in Patients with Malocclusion: A Systematic Review and Meta-Analysis

**DOI:** 10.1155/2021/7751516

**Published:** 2021-09-18

**Authors:** SangYoun Moon, Abdelrahman Magdi Ahmd Mohamed, YaLi He, WenJie Dong, Chen Yaosen, Yan Yang

**Affiliations:** Orthodontic Section, Stomatology Department, Zhongnan Hospital, Wuhan University, Wuhan, China

## Abstract

**Objectives:**

We aimed to summarize the current evidence regarding the impact of extraction vs. nonextraction in orthodontic treatment on patients' soft-tissue profile with malocclusion.

**Methods:**

Between April 30^th^ and November 30^th^, 2020, we searched PubMed and SCOPUS for published papers from inception to November 2020 using “orthodontic,” “extraction,” “nonextraction,” and “Malocclusion.” Included studies were summarized, and relevant data were extracted and analyzed using Review Manager 5.4.

**Results:**

Pooled data from four controlled trials demonstrated a nonsignificant difference between extraction and nonextraction in terms of SNA (MD = 0.50, 95% CI: -0.37, 1.38; *p* = 0.26), SNB (MD = 0.11, 95% CI: -1.23, 1.44; *p* = 0.88), FMA (MD = 1.82, 95% CI: -2.39, 6.02; *p* = 0.40), IMPA (MD = 0.06, 95% CI: -8.83, -8.94; *p* = 0.99), overjet (MD = −1.47, 95% CI: -6.21, 3.26; *p* = 0.54), and overbite (MD = 0.50, 95% CI: -1.40, 2.40; *p* = 0.60). On the other hand, the extraction method significantly increased the ANB compared with the nonextraction group (MD = 0.78, 95% CI: 0.25, 1.31; *p* = 0.004).

**Conclusion:**

The current evidence demonstrated that nonextraction protocols for orthodontic treatment are a safe and effective alternative to extraction protocols; individually tailored treatment strategies should be applied. More randomized controlled trials are critically needed to safely make an evidence-based treatment conclusion.

## 1. Introduction

Malocclusion seems to be a frequent dental anomaly that typically develops during childhood as a dental malalignment or inappropriate relationship with the dental arches [1]. Many problems can result from malocclusion including lack of satisfaction with facial appearance, mastication problems, temporomandibular joint dysfunction, swallowing and speech problems, and dental caries development [2].

The patient profile and aesthetics may be affected by orthodontic treatment. Premolar extractions have been suggested to lead to an undesirable flattened facial appearance more than nonextraction treatment [3–5]. While this has been contested in multiple studies [6–9], controversy is still present regarding the role of premolar extraction treatment on soft-tissue changes in class II malocclusion treatment [4, 10].

Many factors influence the decision to choose between the extraction and nonextraction treatments. For example, clinicians with more orthodontic experience tend to choose the extraction option [11]. Extraction treatment is also preferred in cases with class II malocclusion, open-bite problems, and moderate to severe crowding [12]. It is important to consider that extraction treatment is associated with varying degrees of impact on various vertical dimension outcomes, treatment stability, arch widths, perioral soft tissues, and, subsequently, facial profile [13, 14]. Conversely, nonextraction treatment is generally reserved for minor skeletal and moderate dental discrepancies and crowding.

Multiple previous systematic reviews have tried to address this controversy by combining comparative studies of extraction vs. nonextraction effect on mainly cephalometric perioral soft-tissue changes and facial profile [15–18]. Two of these did not perform quantitative synthesis [15, 16]. Simultaneously, the other two either included mostly potentially biased nonrandomized trials with considerable unresolved heterogeneity [17] or had significant flaws in methodology and a very low number of included studies [18].

Admittedly, the literature's available evidence is hard to combine and analyze quantitatively since most comparative studies are nonrandomized studies. This is understandable since randomized clinical trials (RCTs)—considered the epitome of clinical research—in orthodontics present serious ethical challenges and have received much criticism [19].

In a metaepidemiologic study, orthodontic intervention outcomes seem to be overstated in non-RCTs compared with RCTs and in retrospective studies compared to prospective studies [20]. This evidence suggests that the nature of the clinical intervention trials may affect estimated treatment effects and that the previous meta-analysis findings on this topic are highly questionable.

In this review, we aim to avoid as much as possible the pitfalls of previous meta-analyses by including only prospective controlled trials reporting on the effect of orthodontic extraction vs. nonextraction on cephalometric outcomes of the facial profile.

## 2. Methods

During the preparation of this review, the Cochrane Handbook guidelines of Systematic Reviews and Meta-analysis and the Preferred Reporting Items of Systematic Reviews and Meta-Analysis (PRISMA) were followed [21, 22].

### 2.1. Eligibility Criteria

We included the studies that met our eligibility criteria: (1) studies including patients who were treated either by extraction or nonextraction methods, (2) studies that report data about the dental and soft-tissue changes in response to the treatment, and (3) studies that were experimental in design (RCT, CT, or quasiexperimental). We excluded case reports, animal studies, conference abstracts, and non-English language reviews.

### 2.2. Information Source and Literature Search

Between April 30^th^ and November 30^th^, 2020, we searched PubMed and SCOPUS for published papers from inception to November 2020 using these keywords: “orthodontic,” “extraction,” “non-extraction,” and “Malocclusion.”

### 2.3. Study Selection

The screening process was performed in two steps: (1) title and abstract screening and (2) full-text screening. Both steps were conducted using an offline 2016 Microsoft Excel sheet by two independent reviewers (XX and XX), who assessed the retrieved articles' eligibility. Any disagreement between both reviewers was resolved by a third reviewer (XX).

### 2.4. Extraction of the Relevant Data

We extracted the following domains using an offline data extraction sheet [23]: (1) last name of first author, (2) year of publication, (3) design, (4) population characteristics, (5) sample size, (6) accessible data of studied outcomes, and (7) risk of bias.

### 2.5. Risk of Bias

As described in the Cochrane Handbook for Systematic Reviews of Interventions, we assessed the risk of bias using the Cochrane risk of bias tool (ROB) [24]. Seven domains were evaluated during this step: (1) selection bias, (2) performance bias, (3) detection bias, (4) attrition bias, (5) reporting bias, and (7) other potential sources of bias. The final judgment of the authors was categorized as low, unclear, or high risk of bias.

### 2.6. Assessment of Heterogeneity

To assess the heterogeneity, we used two methods: (1) visual inspection of the forest plots and (2) using the *I*-square (*I*^2^) and chi-square (chi^2^) tests. According to the Cochrane handbook, the interpretation of the *I*^2^ test should be based on the following cutoff points: minimal (0% to 30%), moderate (30% to 60%), and high (60% to 100%).

### 2.7. Data Analysis

We used the Review Manager 5.4 software (Windows version) to analyze the mean difference (MD) and its standard deviation (SD) between the extraction and nonextraction groups. The DerSimonian−Laird fixed-andrandom-effect models were applied. We conducted the analysis of homogeneous data under the fixed-effect model, while heterogeneous data were analyzed under the random-effect model. We analyzed the available data regarding the following outcomes: SNA, SNB, ANB, FAM, IMPA, overjet, overbite, nasolabial angle, Ls-E-plane, and Li-E-plane. Table 1 presents the reference of each outcome. The sequential algorithm was used to perform the sensitivity analysis.

## 3. Results

### 3.1. Literature Searching

**Figure 1:**
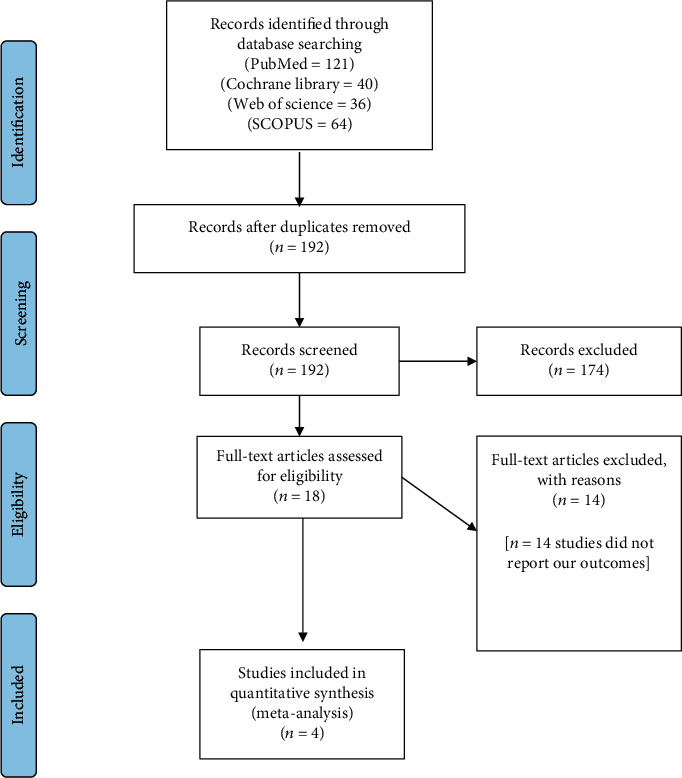
The PRISMA flow diagram.

We have identified 261 documents through the literature search. As results, five studies were included in the systematic review and meta-analysis with a total of 140 participants (70 extracted, 70 nonextracted, and 20 surgical).  Figure 1 shows the screening, inclusion, and exclusion processes.

### 3.2. Characteristics and Baseline Summary

**Figure 2:**
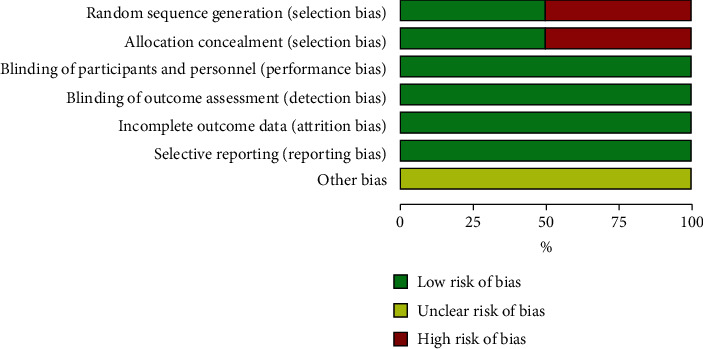
Summary of the risk of bias of included studies.

Two studies were randomized controlled trials, one quasiexperimental and one nonrandomized controlled trial. The mean age of the included patients ranged between 14 and 25.7 years. The majority of included patients were female (75.7%). Regarding the class of malocclusion, class I was reported in two studies: Germeç 2008 (100%) and Khan 2010 (70.5%), while three studies included patients with class II/I: Hemmatpour 2016 (100%), Khan 2010 (29.5%), and Kinzinger 2009 (100%). Two studies extracted four premolars, one study extracted maxillary premolars, and one study extracted one premolar in each quadrant of the upper arch.  Table 2 shows a summary of the included studies.  Figure 2 summarizes the risk of bias of included studies.

### 3.3. SNA

**Figure 3:**
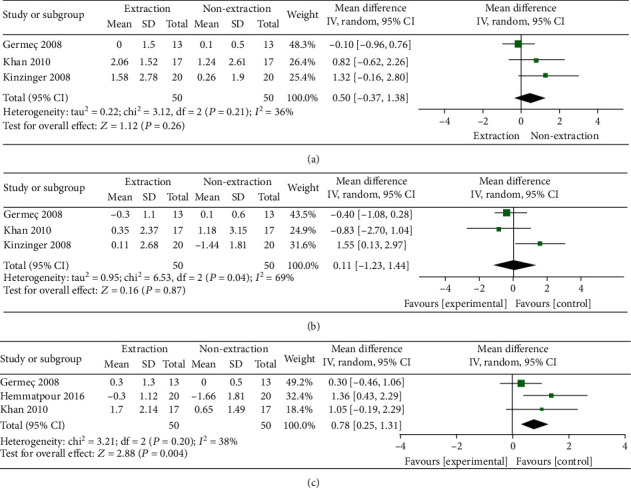
(a–c) The heterogeneity and test for overall effect.

Pooled data of three studies showed nonsignificant difference between the extraction and nonextraction groups in terms of SNA (MD = 0.50, 95% CI: -0.37, 1.38; *p* = 0.26). Pooled data were homogenous (*I*^2^ = 36%; *p* = 0.21), Figure 3(a).

### 3.4. SNB

Overall effect estimate of three studies showed that there was no significant difference between both extraction and nonextraction groups regarding the SNB (MD = 0.11, 95% CI: -1.23, 1.44; *p* = 0.88). Pooled data were heterogeneous (*I*^2^ = 69%; *p* = 0.04). Heterogeneity can be solved by excluding Kinzinger 2008 (*I*^2^ = 0%; *p* = 0.67). After solving the heterogeneity, the overall effect estimate remained nonsignificant (MD = −0.45, 95% CI: -1.09, 0.19; *p* = 0.17) (Figure 3(b)**)**.

### 3.5. ANB

Overall fixed-effect estimate demonstrated that the extraction method significantly increased the ANB compared with the nonextraction group (MD = 0.78, 95% CI: 0.25, 1.31; *p* = 0.004). Pooled data were homogenous (*I*^2^ = 38%; *p* = 0.20), Figure 3(c).

### 3.6. FMA

**Figure 4:**
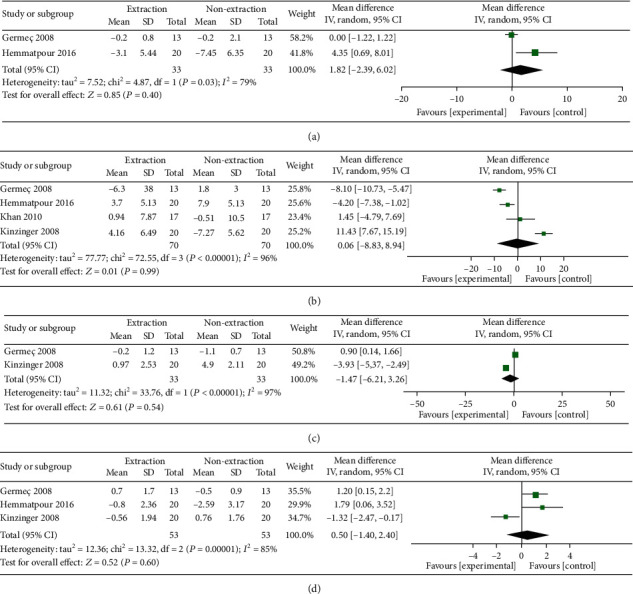
(a–d) The heterogeneity and test for overall effect.

Pooled data of two studies showed nonsignificant difference between the extraction and nonextraction groups in terms of FMA (MD = 1.82, 95% CI: -2.39, 6.02; *p* = 0.40). Pooled data were heterogeneous (*I*^2^ = 79%; *p* = 0.03); however, sensitivity analysis was not applicable, Figure 4(a).

### 3.7. IMPA

Overall random-effect estimate demonstrated that both extraction and nonextraction methods were comparable in terms of IMPA (MD = 0.06, 95% CI: -8.83, -8.94; *p* = 0.99). Pooled data were heterogeneous (*I*^2^ = 96%; *p* < 0.00001). Heterogeneity was best resolved by removing Germeç 2008 and Kinzinger 2008 from the analysis (*I*^2^ = 60%; *p* = 0.11). The overall effect estimate remained nonsignificant (MD = −2.04, 95% CI: -7.42, 3.34; *p* = 0.46), Figure 4(b).

### 3.8. Overjet

Pooled data of two studies showed nonsignificant differences between both groups in terms of overjet (MD = −1.47, 95% CI: -6.21, 3.26; *p* = 0.54). Pooled data were heterogeneous (*I*^2^ = 97%; *p* < 0.00001). Heterogeneity cannot be resolved by sensitivity analysis (Figure 4(c)**)**.

### 3.9. Overbite

Our analysis demonstrated a nonsignificant difference between both groups in terms of overbite (MD = 0.50, 95% CI: -1.40, 2.40; *p* = 0.60). Pooled data were heterogeneous (*I*^2^ = 85%; *p* = 0.001). Heterogeneity can be resolved by excluding Kinzinger 2008 from the analysis (*I*^2^ = 0%; *p* = 0.57). After the sensitivity analysis application, the overall effect estimate showed that the extraction method significantly increased the overbite (MD = 1.36, 95% CI: 0.46, 2.25; *p* = 0.003), Figure 4(d).

### 3.10. Nasolabial Angle

**Figure 5:**
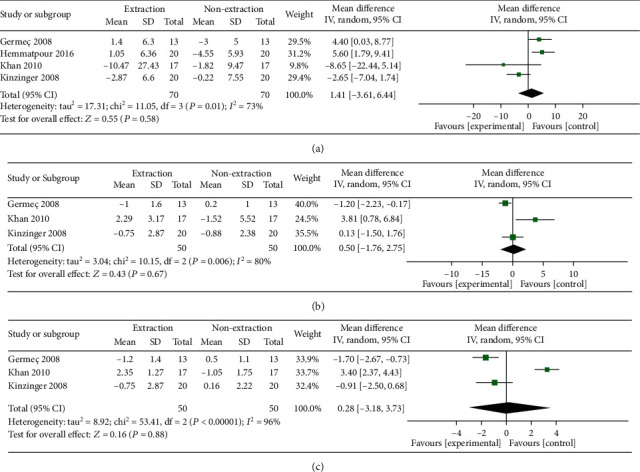
(a–c) The heterogeneity and test for overall effect.

Overall random-effect estimate demonstrated that both extraction and nonextraction methods were comparable in terms of nasolabial angle (MD = 1.41, 95% CI: -3.61, 6.44; *p* = 0.58). Pooled data were heterogeneous (*I*^2^ = 73%; *p* = 0.01). Heterogeneity was best resolved by removing Kinzinger 2008 from the analysis (*I*^2^ = 48%; *p* = 0.15). The overall effect estimate remained nonsignificant (MD = 3.78, 95% CI: -0.68, 8.24; *p* = 0.10) (Figure 5(a)**)**.

### 3.11. Ls-E-Plane

Pooled data of three studies showed nonsignificant difference between both groups in terms of Ls-E-plane (MD = 0.50, 95% CI: -1.76, 2.75; *p* = 0.67). Pooled data were heterogeneous (*I*^2^ = 80%; *p* = 0.006). Heterogeneity can be resolved by excluding Khan 2010 from the analysis (*I*^2^ = 45%; *p* = 0.18); however, the overall effect estimate remained nonsignificant (MD = −0.69, 95% CI: -1.96, 0.57; *p* = 0.28) (Figure 5(b)).

### 3.12. Li-E-Plane

Pooled data of three studies showed nonsignificant difference between both groups in terms of Li-E-plane (MD = 0.28, 95% CI: -3.18, 3.73; *p* = 0.88). Pooled data were heterogeneous (*I*^2^ = 98%; *p* < 0.00001). Heterogeneity can be resolved by excluding Khan 2010 from the analysis (*I*^2^ = 0%; *p* = 0.41), showing a significant reduction in the Li-E-plane within the extraction group (MD = −1.49, 95% CI: -2.31, -0.66; *p* = 0.0004) (Figure 5(c)).

### 3.13. Results of Individual Studies

Kinzinger and his colleagues [25] reported a nonsignificant (*p* = 1.00) difference between the extraction and nonextraction groups in terms of N′-Pn-Pog′ (−0.75 ± 3.98 vs. −0.79 ± 3.28, respectively) and N′-Sn-Pog′ (−1.04 ± 5.26 vs. −1.24 ± 4.22, respectively). Similarly, their findings showed that the effect of both groups was comparable (*p* > 0.05) in the following outcomes: Pog′-Sn on FH, Me′-FH, Sn-FH, Ls-FH, Li-FH, Ls-ML, Li-ML, and Sn-ML.

In the study of Hemmatpour et al. [26], the authors demonstrated that the nonextraction method significantly increased the N′-Gn′ (*p* = 0.029), N′NsPog′ (*n* = 0.002), Pog-Pog′ (*p* = 0.03), and SS-Ls (*p* = 0.001), compared to the extraction group.

Germeç and Taner [3] reported that there was no significant difference between the extraction and nonextraction groups in terms of Ls and Li thickness (*p* = 0.64 and *p* = 0.83), respectively. Likewise, the superior and inferior sulcus depths were similar in both groups (*p* = 0.83 and *p* = 0.22), respectively. Their findings also showed that both groups reduced the maxillary and mandibular sulcus contour with no significant difference (*p* = 0.36 and *p* = 0.41). On the other hand, the Ls-PTV, Li-PTV, and Ls-SnPog′ were significantly (*p* < 0.05) reduced in the extraction group compared to the nonextraction group. Similar to these findings, Khan and Fida [27] found that extraction was associated with a significant reduction in the upper and the lower lip procumbency (*p* = 0.004 and 0.021). However, they showed that there was no significant (*p* > 0.05) difference between both groups in terms of Ls thickness and nasolabial and mentolabial angles.

## 4. Discussion

In this review, we compiled evidence from clinical trials about cephalometric parameters' changes following extraction vs. nonextraction protocols in orthodontic fixation treatment of malocclusion. Four studies were included: two randomized clinical trials, one quasiexperimental trial, and one nonrandomized trial.

While the overall evidence from these studies did not show a statistically significant difference between extraction and nonextraction in our defined outcomes, it has demonstrated that nonextraction is a less interventional, safe, and similarly effective alternative to extraction in malocclusion patients [28]. This is evidenced by cephalometric analysis parameters (SNA, SNB, FAM, IMPA, overjet, overbite, nasolabial angle, Ls-E-plane, and Li-E-plane), which were similar in extraction and nonextraction. It is necessary to keep in mind that the treatment's overall facial attractiveness is more important than final cephalometric values [29]. Accordingly, this consideration was in agreement with our results since multiple studies demonstrated that extraction was not superior to nonextraction in terms of long-term facial aesthetics, age appearance, and soft-tissue measures [30–32].

Our results were also in agreement with Zierhut et al., who demonstrated that the facial profiles of patients with class II division 1 malocclusion who were successfully treated with extraction and nonextraction treatments were similar [33]. The authors also concluded that the facial profile flattening during treatment and long-termfollow-up was primarily due to maturational changes and was not influenced by whether teeth were removed [33].

Extraction treatment is associated with multiple adverse effects, which it may share with nonextraction treatment with various degrees of difference. Extraction is associated with less support for the upper lip after extraction with a flattened, less attractive facial profile [34]. Also, extraction leaves less space for the tongue as the arches become smaller, forcing the tongue to take a backward position, leading to airway blockage and mouth breathing problems [35]. Extraction treatment has also been associated with a higher risk for sleep apnea with its many complications due to restricted space [36]; however, this has been contested [37]. More significantly, extraction has been variably linked to chronic pain and functional bite issues resulting from retraction [38–41].

In a study of 16 orthodontic female patients treated with extraction of premolars, the authors reported that about 12 percent of their sample finished their treatment with a more retrusive facial profile, and if a strict interpretation of numbers had been applied, this percentage would rise to 62% [42]. Furthermore, extraction protocols have been associated with narrower airway size. Recent studies have pointed out that extraction affected the velopharyngeal, glossopharyngeal, hypopharyngeal, and hyoid position and that the velopharyngeal, glossopharyngeal, and hypopharyngeal airway became narrower following orthodontic therapy [43, 44]. Although extraction treatment has been suggested to help with facial height reduction, some studies have contested this. It has been shown that the bicuspid extraction technique for facial height reduction does not provide any statistically significant changes for patients after treatment [45, 46].

A case for extraction treatment could be argued, however, for a special subset of patients. One study showed that extraction treatment commonly produces positive results for patients where the objective is to reduce lip procumbent [47]. Another study showed that premolar extractions positively influenced the developing maxillary third molar angulations, and these improved angulations could favor third molar eruptions later in life [48]. Combined with the potentially negative aspects of many nonextraction treatment types (such as instability, procumbent, and inefficiency), careful choice of evidence-based treatment decisions should be considered.

Although our study managed to avoid the bias included in previous meta-analyses, which compiled data from retrospective, nonrandomized trials, our study is still limited by the small number of studies included and the subsequently small sample size. The difference between the endpoints of our study and previous meta-analyses makes a comparison of outcomes questionable. As shown previously, both treatment protocols did not differ, and the implications of our results were similar. Although addressed by removing one or more studies during quantitative synthesis, heterogeneity should be considered during clinical decision-making. Moreover, the trial by Kinzinger et al. was nonrandomized and is subject to selection bias.

## 5. Conclusion

The current evidence demonstrated that the extraction method might be associated with some soft-tissue benefits in case of a convex profile with acute nasolabial angle; however, nonextraction protocols for orthodontic treatment are a safe and effective alternative to extraction protocols; individually tailored treatment strategies should be applied. More randomized controlled trials are critically needed to safely make an evidence-based treatment conclusion.

## Figures and Tables

**Table 1:** Reference of studied outcomes.

Outcome	Index
SNA	The angle between the anterior cranial base and the deepest concavity of the maxilla's anterior contour
SNB	The angle between the anterior cranial base and the deepest concavity of the mandible's anterior contour
N	Most anterior point of the frontonasal suture in the midsagittal plane
A	The most posterior point in the concavity between the anterior nasal spine and the dental alveolus
B	The most posterior point in the concavity along the anterior border of the symphysis
ANB	The angle formed by NA and NB
FMA	The angle formed by Frankfort horizontal plane and mandibular plane
IMPA	The angle formed by the axial inclination of the mandibular incisor and the mandibular plane
Overjet	Distance between U1i (tip of the maxillary central incisor) and L1i (tip of the mandibular central incisor) in the horizontal plane
Overbite	Distance between U1i (tip of the maxillary central incisor) and L1i (tip of the mandibular central incisor) in the vertical plane
Nasolabial angle	The angle formed by columella tangent and Sn- (point at the junction of the columella and upper lip) Ls (most anterior point on the curve of the upper lip) line
Ls-E-plane	Distance from upper lip to the E-line
Li-E-plane	Distance from lower lip to the E-line
N′-Pn-Pog′	The angle formed by soft-tissue nasion, nose tip, and soft-tissue pogonion
N′-Sn-Pog′	The angle formed by soft-tissue nasion, subnasale, and Pog′
Pog′-Sn on FH	The linear distance between the Pog′ and the subnasale as projected onto the Frankfort horizontal
Me′-FH	The vertical distance between soft-tissue menton and FH
Sn-FH	The vertical distance between subnasale and FH
Ls-FH	The vertical distance between the upper lip and FH
Li-FH	The vertical distance between the lower lip and FH
Ls-ML	Vertical distance between Ls and mandibular line
Li-ML	The vertical distance between Li and mandibular line
Sn-ML	The vertical distance between subnasale and mandibular line
Pog-Pog′	Chin thickness
N′NsPog′	The angle formed by soft-tissue nasion and Pog′
Ls-PTV	Distance between the pterygoid vertical plane and Ls
Li-PTV	Distance between the pterygoid vertical plane and li
Ls-SnPog′	The distance formed by upper lip, subnasale, and Pog′

**Table 2:** Characteristics of included studies and patients.

Study ID	Study design	Groups	Included patients	Female (*n*)	Age (mean ± SD)	Class I angles	Class II/I angles	Extracted tissue	Studied outcomes	Conclusion
Germeç 2008	RCT	Extraction (*n* = 13)	Patients with moderate dental arch crowding and balanced facial profiles and dentoskeletal relationships	11	18.1 ± 3.7	26 (100%)	0	Four premolars	Skeletal, dental, and soft tissue	Both extraction and nonextraction provide comparable outcomes with good facial profile and moderate dental crowding
		Nonextraction (*n* = 13)		11	17.8 ± 2.4					

Hemmatpour 2016	RCT	Extraction (*n* = 20)	Patients with 12–18 years old, having permanent dentition only, being at stages 4–6 of cervical vertebral maturation index (CVMI S4–6), having a molar full-cusp class II relationship, having ANB angles C 4, upper-incisor-to-NA-line angles above 18, having full-cusp molar class II, and being clinically proper candidates for upper premolar extraction or fixed functional therapy	12	15.40 ± 0.99	0	40 (100%)	Maxillary premolar	Skeletal, dental, and soft tissue	Extraction reduced the interincisal angle and protruded the lower incisors
		Nonextraction (*n* = 20)		13	15.75 ± 1.02					

Khan 2010	Quasiexperimental	Extraction (*n* = 17)	Patients having undergone routine orthodontic treatment	13	14 years and 6 months	24 (70.5%)	10 (29.5%)	Four premolars	Incisal and soft-tissue effects	Both extraction and nonextraction provide comparable outcomes
		Nonextraction (*n* = 17)		13	14 years and 8 months					

Kinzinger 2009	CT	Extraction (*n* = 20)	Young adults presenting a skeletal class II, division 1 malocclusion	33	18.7 ± 2.4	0	60 (100%)	One premolar in each quadrant of the upper arch	Skeletal, dental, and soft tissue	Fixed functional appliances are a treatment alternative to extraction therapy but to a lesser extent to orthognathic surgery
		Nonextraction (*n* = 20)			17.6 ± 2.3					
		Surgical group (*n* = 20)			25.7 ± 5.4					

RCT: randomized control trial; SD: standard deviation; CT: clinical trial; *n*: number.

## Data Availability

Data will be available when requested from the corresponding author.

## Abbreviations

RCTs: Randomized control trials
SD: Stander deviation
CT: Clinical trial
CI %: Percentage of the confidence interval
*n*: Number
ROB: Risk of bias
MD: Mean difference
*p*: Value of probability
SNA: The angle between the anterior cranial base and the deepest concavity of the maxilla's anterior contour
SNB: The angle between the anterior cranial base and the deepest concavity of the mandible's anterior contour
N: Most anterior point of the frontonasal suture in the midsagittal plane
A: The most posterior point in the concavity between the anterior nasal spine and the dental alveolus
B: The most posterior point in the concavity along the anterior border of the symphysis
ANB: The angle formed by NA and NB
FMA: The angle formed by the Frankfort horizontal plane and mandibular plane
IMPA: The angle formed by the axial inclination of the mandibular incisor and the mandibular plane
Overjet: Distance between U1i (tip of the maxillary central incisor) and L1i (tip of the mandibular central incisor) in the horizontal plane
Overbite: Distance between U1i (tip of the maxillary central incisor) and L1i (tip of the mandibular central incisor) in the vertical plane
Nasolabial angle: The angle formed by columella tangent and Sn- (point at the junction of the columella and upper lip) Ls (most anterior point on the curve of the upper lip) line
Ls-E-plane: Distance from upper lip to the E-line
Li-E-plane: Distance from lower lip to the E-line
N′-Pn-Pog′: The angle formed by soft-tissue nasion, nose tip, and soft-tissue pogonion
N′-Sn-Pog′: The angle formed by soft-tissue nasion, subnasale, and Pog′
Pog′-Sn on FH: The linear distance between the Pog′ and the subnasale as projected onto the Frankfort horizontal
Me′-FH: The vertical distance between soft-tissue menton and FH
Sn-FH: The vertical distance between subnasale and FH
Ls-FH: The vertical distance between the upper lip and FH
Li-FH: The vertical distance between the lower lip and FH
Ls-ML: The vertical distance between Ls and mandibular line
Li-ML: The vertical distance between Li and mandibular line
Sn-ML: The vertical distance between subnasale and mandibular line
Pog-Pog′: Chin thickness
N′NsPog′: The angle formed by soft-tissue nasion and Pog′
Ls-PTV: Distance between the pterygoid vertical plane and Ls
Li-PTV: Distance between the pterygoid vertical plane and Li
Ls-SnPog′: The distance formed by the upper lip, subnasale, and Pog′.
